# Synthesis of Novel Hybrid Molecules from Precursors With Known Antiparasitic Activity

**DOI:** 10.3390/molecules14041483

**Published:** 2009-04-09

**Authors:** Haythem A. Saadeh, Ibrahim M. Mosleh, Mohammad S. Mubarak

**Affiliations:** 1 Chemistry Department, Faculty of Science, University of Jordan, Amman 11942, Jordan; 2 Department of Biological Sciences, Faculty of Science, University of Jordan, Amman 11942, Jordan

**Keywords:** Hybrid Molecules, Metronidazole, Chloroquine, *Entamoeba Histolytica*, *Giardia Intestinalis*

## Abstract

Three novel new compounds derived from antiparasitic precursors have been synthesized and tested for their antiamoebic and antigiardial activities. The condensation of 2-(2-methyl-5-1H-nitroimidazolyl)ethylamine (**6**) with 5-nitro-2-furylacrylic acid (**7**) gave 3-(5-nitrofuran-2-yl)-N-[2-(5-nitroimidazol-1-yl)ethyl]acrylamide (**8**). Condensation of **7** with 7-chloro-4-(piperazin-1-yl)quinoline (**9**) afforded 1-[4-(7-chloroquinolin-4-yl)piperazin-1-yl)-3-(5-nitrofuran-2-yl)propenone as a mixture of two isomers; 10-a (the E-isomer) and 10-b (the Z-isomer). In addition, the reaction of 9 with 1-(2-bromoethyl)-2-methyl-5-nitroimidazole (**11**) in the presence of K2CO3 and NaI yielded 7-chloro-4-(4-[2-(5-nitroimidazol-1-yl)ethyl]-piprazin-1-yl)quinoline (**12**). On the basis of preliminary screening data for these new compounds, compound **12** exhibited potent lethal activities against *Entamoeba histolytica* and *Giardia intestinalis*; its IC_50_ ( about 1 µM) was lower, at least by a factor of five, compared to the standard drug, metronidazole. In addition, the IC_50_ of compound **12** against the tested parasites is 600 times below that against Hep-2 and Vero cells. Compounds **8** and **10-a** also exhibited potent or moderate antiamoebic and antigiardial activities with IC_50 values_ of about 5.5 µM, and 140 µM, respectively, against the tested parasites. These two hybrid molecules, **8**, **10-a**, were also non-cytotoxic at the lethal concentrations against the parasites.

## Introduction

The concept of “hybrid drugs” has been gaining popularity in medicine. Since a single drug is not always able to adequately control the illness, the combination of drugs with different pharmaco-therapeutic profile may be needed [1]. Drugs involving the incorporation of two drug pharmacophores in a single molecule with the intention of exerting dual drug action have been described [2]. For example, one of the hybrid parts may be incorporated to counterbalance the known side effects associated with the other hybrid part, or to amplify its effects through action on another biological target. Ultimately, no matter how familiar the building blocks may be, hybrid drug molecules may, at their core, become new molecules with identities independent of their precursors.

Encouraging examples of hybrid drug use on systemic heart disease and malaria were recently published in the literature. Bisi and coworkers [1] reported the synthesis and pharmacological profile of some hybrid compounds bearing both the benzazepinone moiety present in Zatebradine (a drug that is used to reduce the heart rate without concomitant negative inotropic or hypertensive effects) and typical β-blocker aryloxypropanolamine groups; the new compounds proved to be endowed with negative chronotropic and inotropic activity and are weak vasorelaxant agents.

Chloroquine (**1**) had been used as the prime therapy for treating malaria for nearly half a century [3], but *Plasmodium falciparum*, the cause of the most deadly variety of malaria, has now become chloroquine resistant in all malaria-endemic regions of the globe [3]. Some strains have also developed resistance to mefloquine (**2**) [4] and even to the naturally occurring and highly efficient antimalarial quinine (**3**) [3,5]. 
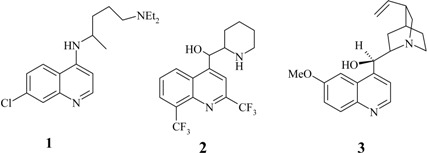


One of the generally accepted options to inhibit or delay the emergence of resistance to new antimalarial agents is the combination therapy [2,4,6]. The principle of combination drug therapy can be achieved by either using concomitant administration of two or more single active drugs or by drugs in which the single active agents are combined in one molecule, i.e., hybrid molecules; the two drug-like portions (pharmacophores) have independent modes of action that make the emergence of drug resistance less likely to occur.

Recently, Peyton and his team [3] synthesized a class of hybrid molecules termed “reversed chloroquines”; these drugs were found to be effective against both chloroquine-sensitive and chloroquine-resistant strains of *Plasmodium falciparum*. In their work, they combined chloroquine with the so-called reversal agent **4**, which counters resistance by inhibiting a membrane channel that pumps chloroquine out of the parasite's digestive vacuole, the site at which the drug acts against the parasite. The best of Peyton's hybrid drugs were about 10 times more effective against drug-sensitive malaria than chloroquine itself. Adding to this hybrid's promise is its ability to kill a chloroquine-resistant strain of *P. falciparum*. More recently, Walsh and coworkers [7] combined fast-acting artemisinin and slow-acting quinine into a hybrid drug **5** for malaria, for which drug resistance is a barrier to effective treatment. *In vitro* assays showed that the hybrid is more effective against drug-sensitive and drug-resistant malaria than the individual drugs alone or a cocktail made of a 1:1 molar ratio of the two. Walsh suggested that the hybrid drug may increase cellular uptake which improves the treatment's efficacy. 
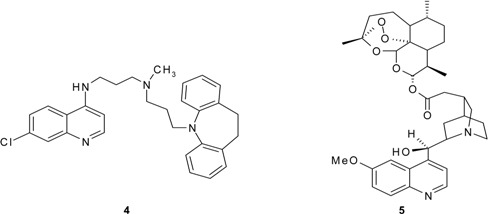


**Scheme 1:**
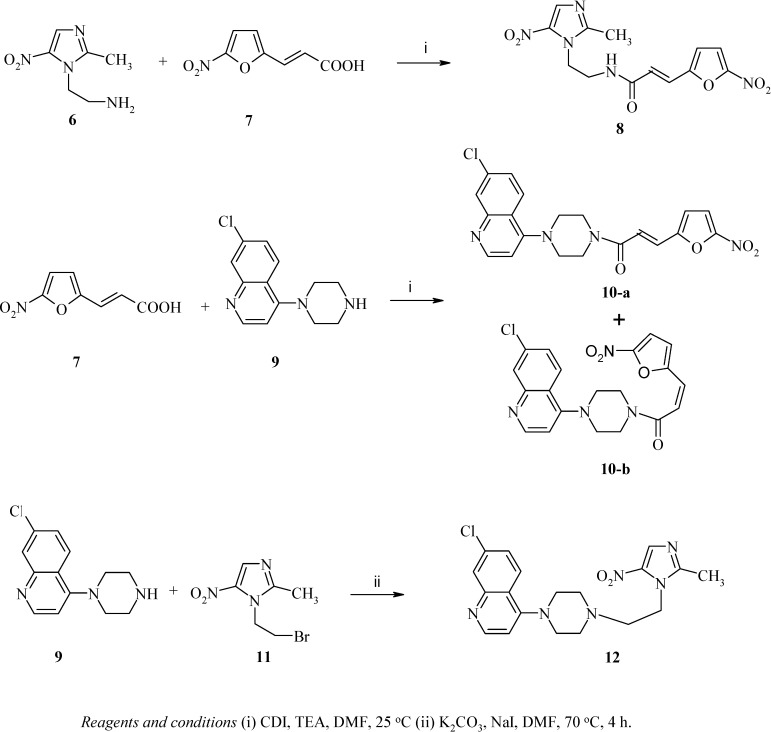
Preparation of hybrid molecules **8**, **10-a**, **10-b** and **12**.

As part of our ongoing research in the synthesis of new compounds of pharmacological interest [8,9,10,11], we describe herein the synthesis, characterization, and antiamoebic and antigiardial activities of three new hybrid molecules assembled from precursors with known antiparasitic activity, namely, metronidazole (Flagyl), chloroquine (CQ), and 5-nitrofuranacrylic acid (Scheme 1). The three hybrid molecules displayed good antiamoebic and antigiardial activity *in vitro*; one exhibited higher activity than the standard drug, metronidazole.

## Results and Discussion

### Chemistry

Syntheses of the hybrid molecules **8**, **10-a**, **10-b**, and **12** were carried out via the route shown in Scheme 1. Condensation of 2-(2-methyl-5-1H-nitroimidazolyl)ethylamine (**6**) with **7** in DMF using carbonyl diimidazole (CDI) as a coupling agent and in the presence of triethylamine gave the desired compound **8** as the *E*-isomer, as confirmed by its ^1^H-NMR spectrum where the coupling constant, *J*, of the vinylic protons resonating at *δ* 6.62 and 7.28 ppm was 15.7 Hz; the *Z*-isomer was not detected. The ^1^H-NMR and ^13^C-NMR spectra of 8 confirmed the formation of an amide bond; the corresponding proton appeared at *δ* 8.58 ppm and the amide carbonyl carbon at 164.7 ppm. In addition, the presence of N-H, C=O (amide), and conjugated C=C absorption bands in the IR spectrum of **8** at 3362, 1694 and 1605 cm^-1^, respectively, confirmed that compound **8** was obtained. Condensation of **7** with **9** in DMF, using CDI as a coupling reagent, and in the presence of triethylamine afforded **10-a** (the *E*-isomer) and **10-b** (the *Z*-isomer) as a mixture of two isomers with identical M^+^ and elemental analysis. The ^1^H-NMR and ^13^C-NMR spectra suggested an *E*-*Z* isomerization of the double bond during the course of the reaction, with the *E*-isomer being the major product. The ^1^H-NMR spectra of the products confirmed their stereochemistry. The vinylic protons of the double bond at *δ* 6.66 and 7.30 ppm couple most strongly, with a *J-* value of about 15.7 Hz, for the *E* isomer, **10-a** and for the Z isomer, **10-b**, a *J*-value of about 7.8 Hz was found for the corresponding vinylic protons at *δ* 6.72 and 7.36 ppm. Furthermore, in **10-b**, the piperazine CH_2_ protons adjacent to the amide bond are not equivalent; that was not the case in **10-a**. The former has two different CH_2_ protons (3.85 and 4.0 ppm) corresponding to two hydrogens each, and two different carbons that resonate at 42.2 and 45.7 ppm, whereas the latter has just one CH_2_ peak at 3.70-3.80 ppm corresponding to four hydrogens and has just one carbon that resonates at 46.2 ppm. The IR spectra of compounds **10a** and **10b**, showed the characteristic C=O, and C=C stretching vibrations at 1676 and 1637 cm^-1^, respectively, in addition to the absence of a N-H absorption band. Alkylation of **9** with 1-(2-bromoethyl)-2-methyl-5-nitroimidazole (**11**) in DMF and in the presence of K_2_CO_3_ and NaI yielded the hybrid molecule **12**.

The ^1^H-NMR and ^13^C-NMR spectra of all prepared compounds and precursors are in total agreement with the suggested structures. DEPT experiments were employed to differentiate secondary and quaternary carbons from primary and tertiary carbons. Additional support of the proposed structures comes from mass spectral data; low resolution or high resolution mass spectra of the prepared compounds showed the correct molecular ions, (M^+·^), as suggested by their molecular formulas or fragments that stem from the molecular ion. Analyses of the molecular ions and the fragmentation pattern are used in the identification and characterization of these compounds. 

### Antiamoebic and antigiardial activity

The antiamoebic and antigiardial activities of the three compounds **8**, **10-a**, and **12** were investigated using *in vitro* bioassays that included their precursors and their combinations as controls. The cytotoxicity of the three novel hybrids on the two cell lines, Hep-2 and Vero cells, was also investigated and compared with that of the standard antiamoebic and antigiardial drug, metronidazole. The IC_50 values _of the compounds against *Entamoeba histolytica*, *Giardia intestinalis*, and the two cell lines are given in Table 1.

**Table 1:** *In vitro* antiamoebic and antigiardial activities of compounds **8**, **10-a**, **12**, their precursors, and the standard drug, metronidazole, and the cytotoxic activity of **8**, **10-a**, **12**, and metronidazole.

Mean IC_50_ ± SD ^(n)^				
(µM)				
Compound	*Entamoeba histolytica*	*Giardia intestinalis*	Hep-2 cells	Vero cells
**8**	5.32 ± 0.21	5.56 ± 0.37	108.46 ± 8.02	90.55 ± 7.83
**10-a**	139.96 ± 6.37	144.82 ± 5.37	574.03 ± 7.77	593.04 ± 8.71
**12**	0.96 ± 0.23	0.97 ± 0.32	604.52 ± 12.69	616.25 ± 16.94
**Metronidazole**	5.20 ± 1.03	5.70 ± 0.78	1460.86 ± 18.31	1495.13 ± 17.79
**6**	18.20 ± 2.28	18.50 ± 1.43		
**7**	94.26 ± 9.76	88.42 ± 7.10		
**9**	55.70± 5.00	67.51 ± 8.67		
**Chloropip/furan**	22.53/22.53 ±1.07	25.75/25.75 ± 2.23		
**Chloropip/metro**	1.04/1.04 ± 0.21	1.17/1.17 ± 0.26		
**Furan/metroamine**	3.19/3.19 ± 0.21	3.77/3.77 ± 0.33		

**6** = 2-(2-Methyl-5-1H-nitroimidazolyl)ethylamine; **7** = 5-Nitro-2-furylacrilic acid; **9** = 7-Chloro-4-(piperazine-1-yl) quinoline; Chloropip/metro = 1:1 mixture of **9** and metronidazole; Furan/ metroamine = 1:1 mixture of **6** and **7**; Chloropip/furan = 1:1 mixture of **7** and **9**; (*n*) = six determinations

As indicated in the table, the three hybrid molecules **8**, **10-a**, and **12** showed biological activities against *Entamoeba* and *Giardia*. Compound **12**, with an IC_50 _of around one micromolar, was the most active against *Entamoeba* and *Giardia* compared with the other compounds. In terms of molar concentration, this hybrid is at least five times more active than metronidazole. In addition, the cytotoxicity of this hybrid molecule against Hep-2 and Vero cells, represented by the IC_50_,was slightly above 600 µM. In other words, the IC_50_ of this compound against the tested parasites is about 600 times less than that against Hep-2 and Vero cells. If the IC_50 _against the parasites and the cell lines are considered, compound **12** is then about two times better than metronidazole with IC_50_ ratio of around 270 times higher. 

Interestingly, the tested compounds exhibited an almost similar pattern of activity against both *G. intestinalis and E. histolytica* (Table 1), indicating that each compound affects both parasites by a similar mechanism of action. In addition, the results in Table 1 reveal that compound **12** is more active than its precursors, 7-chloro-4-(piperazin-1-yl)quinolines (**9**) and metronidazole when tested separately. Additionally, the activity of one molar fraction of **12** is almost the same as the activity of a mixture fraction of about one molar from each of its precursors. The fact that the mixture of the two precursors has a synergistic effect over the standard drug alone actually confirms the boosted activity of the hybrid **12** and the necessity of having the hybrid as a drug candidate. The hybrid molecule **8** is more potent than its precursors (**6** and **7**) and is almost equivalent to metronidazole, but the hybridization turned it relatively more cytotoxic (Table 1). On the other hand, although compound **10-a** remained moderately active, but it is still less potent than any of its precursors (**7** and **9**) or their mixture (Table 1).

The activities exhibited by the three hybrids, especially compound **12**, suggest that the hybrids may be used as new lead compounds in the development of new antiparasitic drugs. Moreover, the importance of such biologically active, non-cytotoxic hybrid molecules, especially **12**, lies in their potential contribution to overcome the problem of resistance of pathogens to the standard drugs. This problem is arising now and may become serious in the future [12,13,14]. The widespread prophylactic use of the standard drugs may support the spread of resistant strains potentially developing through different proposed mechanisms [15,16,17,18]. Therefore, and because of the limited number of drugs available in the market against anaerobic protozoal parasites and bacteria, there is a serious need for new active compounds. The molecular modification on the original drugs, offers alternatives that may bypass the already developed mechanisms adopted by the anaerobic pathogens against the standard drugs. Our three hybrid compounds are good drug candidates to be tested against metronidazole-resistant parasites and bacteria. 

## Experimental

### General

Melting points were measured with a Fischer-Johns melting point apparatus and are uncorrected. Infrared (IR) spectra were recorded as KBr discs on a Nicolet-400 Fourier transform infrared spectrophotometer from 400 to 4000 cm^-1^. All new compounds were analyzed for C, H, and N, and the observed results agreed with the calculated percentages to within ±0.4%. ^1^H- and ^13^C-NMR were obtained with the aid of a Bruker-DPX 300 MHz spectrometer with CDCI_3_ or DMSO-d_6_ as solvents, chemical shifts are reported in δ ppm relative to TMS as internal standard. Solid-probe high-resolution mass spectral data were acquired with the aid of a Thermo Electron Corporation MAT 95XP-Trap instrument, operated in the positive chemical-ionization (CI) mode with methane as the reagent gas. Low resolution mass spectra were measured using a Finnegan MAT TSQ-70 spectrometers at 70 eV (Finnegan MAT, USA); ion source temperature = 200 ºC. Solid-probe high-resolution mass spectral data were acquired with the aid of a Thermo Electron Corporation MAT 95XP-Trap instrument, operated in the positive chemical-ionization (CI) mode with methane as the reagent gas. Elemental analyses were obtained with a Eurovector Euro EA3000, CHNS-O elemental analyzer. Thin-layer chromatography was carried out using glass plates, precoated with silica gel 60 GF_254_, supplied by Fluka and with silica gel plates ((Macherey-Nagel). The following compounds were obtained from Acros Organics and were used without further purification: carbon tetrabromide, *N,N´*-carbonyldiimidazole, 1-(2-aminoethyl)-2-methyl-5-nitroimidazole dihydrochloride monohydrate (**6**), 4,7-dichloro-quinoline, metronidazole, and 2-furylacrylic acid. 5-Nitro-2-furylacrilic acid (**7**) was synthesized and purified according to published procedures [19].

### 1-(2-Bromoethyl)-2-methyl-5-nitro-1-imidazole (**11**)

The title compound was synthesized according to the following general procedure: to a mixture of metronidazole (3.42 g, 20 mmol) and carbon tetrabromide (8.0 g, 30 mmol) in dry THF (20 mL) was added triphenylphosphine (5.8 g, 21 mmol), portionwise over 15 min at 0 ºC and the mixture was stirred for 1.5 h at room temperature. Water was then added and the reaction mixture was extracted with CH_2_Cl_2_ (2 x 20 mL). The organic layer was dried over anhydrous Na_2_SO_4_ and concentrated under reduced pressure, and the residue was purified using column chromatography with CH_2_Cl_2 _-hexane (3:1 v/v), to give pale-yellow crystals (5.43 g, 91%, mp. 81-82 ºC; Lit. mp = 80-81 ºC [20]). ^1^H-NMR (CDCl_3_): *δ* = 2.50 (s, 3H, CH_3_), 3.64 (t, 2H, *J* = 6.1 Hz), 4.61 (t, 2H, *J* = 6.1 Hz), 7.90 (s, 1H). ^13^C-NMR: *δ* = 14.03 (CH_3_), 39.2, 44.6, 132.8, 138.8, 152.1.

### 7-Chloro-4-(piperazine-1-yl) quinoline (**9**)

This compound was synthesized according to published procedures [21] that involved stirring a mixture of piperazine (10.88 g, 126.45 mmol), potassium carbonate (1.05 g, 7.58 mmol), triethylamine (5.28 mL, 37.88 mmol) and 4,7-dichloroquinoline (5.00 g, 25.25 mmol) in *N*-methyl-2-pyrrolidinone (17.7 mL) under nitrogen at 135 °C for 2 h. After cooling to room temperature, the mixture was diluted with CH_2_Cl_2_ (200 mL). The reaction mixture was then washed with brine (2 × 50 mL), dried (MgSO_4_) and concentrated under reduced pressure. The resulting oil was purified by column chromatography on silica gel using CH_2_Cl_2_/MeOH (4:1 v/v) as the eluent to afford the desired product (5.40 g, 86%, mp 112–114; lit. mp. 113-115 °C [21]). ^1^H NMR (CDCl_3_) δ = 8.62 (d, *J* = 5.1 Hz, 1H), 8.04 (d, *J* = 2.1 Hz, 1H), 7.96 (d, *J* = 9.0 Hz, 1H), 7.35 (dd, *J* = 2.1 and 9.0 Hz, 1H), 6.76 (d, *J* = 5.0 Hz, 1H), 3.16-3.20 (m, 4H), 1.65 (s, 1H); ^13^C-NMR (CDCl_3_) δ = 151.9, 150.2, 134.9, 128.9, 126.1, 125.2, 121.9, 108.9, 53.6 (2C), 46.1 (2C).

### 3-(5-nitrofuran-2-yl)-N-[2-(5-nitroimidazol-1-yl)ethyl]acrylamide (**8**) and E-Z isomers of 1-[4-(7-chloroquinolin-4-yl)piperazin-1-yl)]-3-(5-nitrofuran-2-yl)propenone (**10-a** and **10-b**)

To a stirred solution of 5-nitro-2-furylacrylic acid (7, 0.35 g, 1.90 mmol) in DMF (3 mL) under N_2_, was added a solution of *N,N´*-carbonyldiimidazole, CDI, (0.3 g, 1.90 mmol) in DMF (4 mL). After stirring for 15 minutes at room temperature, a solution of the appropriate amine (**6** or **9**) (1.90 mmol) and triethylamine (0.5 mL) in DMF (4 mL) was added and the mixture was stirred for 3 hours under N_2_. After completion of reaction, the mixture was treated with water (20 mL) and extracted with ethyl acetate (2 x 20 mL). The organic layer was washed with water (3 x 30 mL), dried over anhydrous sodium sulfate and the solvent was removed under reduced pressure. The residue was purified by means of thin layer chromatography with chloroform-methanol (95:5 v/v) to afford compounds **8**, **10-a** and **10-b**. The *E* and *Z* isomers, **10-a** and **10-b**, respectively, were separated by preparative thin layer chromatography on silica gel using chloroform-methanol (95:5 v/v) as eluent; the *Z* isomer has a higher R_f_ value than its *E* counterpart.

Compound (**8**): Yield 0.45 g (63%). IR (cm^-1^): 3362 (NH), 1694 (C=O), 1605 (C=C); ^1^H-NMR (DMSO): *δ* = 2.35 (s, 3H), 3.60-3.70 (t, *J* = 5.6 Hz, 2H), 4.30-4.40 (t, *J* = 5.5 Hz, 2H), 6.62, 7.28 (dd, *J*_1_ = 15.7 Hz, *J*_2_ = 2.8 Hz, 2H, C=C trans), 7.05, 7.68 (dd, *J*_1_ = 3.8 Hz, *J*_2_ = 3.8 Hz, 2H, furan), 7.95 (s, 1H), 8.58 (br, NH). ^13^C-NMR (DMSO): *δ* = 14.3 (CH_3_), 38.7 (CH_2_), 45.8 (CH_2_), 115.2 (CH), 116.9 (CH), 125.3 (CH), 125.6 (CH), 133.7 (CH), 139.0 (C), 151.9 (C), 152.1 (C), 153.5 (C), 164.7 (C). EIMS (probe) 70 eV, *m/z* (rel. int.): 336 [M+1]^+^ (9), 289 [M-46]^+^ (67), 166 [M-169]^+^ (100); Anal. Calcd. for (C_13_H_13_N_5_O_6_): C, 46.57; H, 3.91; N, 20.89. Found: C, 46.63; H, 3.93; N, 20.68.

Compound (**10-a**): Yield 0.45 g (56%). IR (cm^-1^): 1676 (C=O), 1637 (C=C); ^1^H-NMR (DMSO): 3.20-3.30 (m, 4H), 3.70-3.80 (m, 4H), 6.66, 7.30 (dd, *J*_1_ = 15.7 Hz, *J*_2_ = 2.8 Hz, 2H, C=C trans), 6. 78 (d, *J* = 3.8 Hz, 1H), 6.86, 7.68 (dd, *J*_1_ = 3.8 Hz, *J*_2_ = 3.8 Hz, 2H, furan), 7.44 (dd, *J*_1_= 2.1 Hz, *J*_2_ = 9.0 Hz, 1H), 7.96 (d, *J* = 9.0 Hz, 1H), 8.04 (d, *J* = 2.1 Hz, 1H), 8.70 (d, *J* = 4.70 Hz, 1H). ^13^C-NMR (DMSO): *δ* 46.2 (CH_2_), 51.7 (CH_2_), 110.3 (CH), 119.2 (CH), 121.8 (C), 126.5 (CH), 128.6 (CH), 129.4 (CH), 134.2 (C), 137.7 (CH), 150.1 (C), 150.9 (C), 152.8 (CH), 156.3 (C), 163.7 (C). HRMS (CI) *m/z*: calcd for C_20_H_18_ClN_4_O_4_ [M + H]^+^ 413.1004, found 413.1011. 

Compound (**10-b**): Yield 0.12 g (15%). IR (cm^-1^): IR (cm^-1^): 1672 (C=O), 1628 (C=C); ^1^H-NMR (DMSO): 3.10-3.20 (m, 4H), 3.85 (t, *J* = 3.7 Hz, 2H), 4.0 (t, *J* = 3.7 Hz, 2H), 6.72, 7.36 (dd, *J*_1_ = 7.8 Hz, *J*_2_ = 2.6 Hz, 2H, C=C cis), 6.88 (d, *J* = 3.60 Hz, 1H), 6.82, 7.72 (dd, *J*_1_ = 3.60 Hz, 2H, *J*_2_ = 3.8 Hz, 2H, furane), 7.42 (dd, *J*_1_ = 2.1 Hz, *J*_2_ = 9.0 Hz, 1H), 7.93 (s, 1H), 8.05 (d, *J* = 8.9 Hz, 1H), 8.68 (d, *J* = 4.7 Hz, 1H). ^13^C-NMR (DMSO): *δ* 42.3 (CH_2_), 45.7 (CH_2_), 52.1 (CH_2_), 52.6 (CH_2_), 110.2 (CH), 115.4 (CH), 116.4 (CH), 121.9 (C), 122.6 (CH), 126.4 (CH), 126.5 (C), 127.8 (CH), 128.6 (CH), 134.2 (C), 150.1 (C), 152.1 (C), 152.7 (CH), 154.2 (C), 156.4 (C), 163.7 (C). EIMS (probe) 70 eV, *m/z* (rel. int.): 414 [M+2]^+^ (10), 412 [M]^+^ (24), 248 [M-166]^+^ (11), 246 [M-166]^+^ (34), 219 [M-195]^+^ (32), 217 [M-195]^+^ (100); Elemental anal. calcd. (%) for C_20_H_17_ClN_4_O_4_: C, 58.19; H, 4.15; N, 13.57. Found (%): C, 58.01; H, 4.27; N, 13.34.

### Synthesis of 7-chloro-4-(4-[2-(5-nitroimidazol-1-yl)ethyl]piperazin-1-yl)quinoline (**12**)

A mixture of 7-chloro-4-(piperazin-1-yl)quinolines (**9**, 0.3 g, 1.3 mmol), 1-(2-bromoethyl)-2-methyl-5-nitroimidazole (**11**, 0.23 g, 1.3 mmol), K_2_CO_3_ (0.21 g, 1.4 mmol) and NaI (0.25 g, 1.4 mmol) in DMF (5 mL) was heated at 70 ºC for 4 h. The reaction mixture was cooled, treated with water, and extracted with CHCl_3_. The organic layer was dried over anhydrous sodium sulfate and concentrated under reduced pressure. The crude product was purified by silica gel plates using ethyl acetate-hexane (3:1 v/v) to give the desired product as off white crystals. Yield 0.20 g (46%); mp 58-61 ºC. ^1^H-NMR (DMSO): *δ* 2.46 (s, 3H), 2.70-2.80 (m, 6H), 3.10-3.20 (m, 4H) 4.40 (t, *J* = 5.5 Hz, 2H), 6.75 (d, *J* = 4.3 Hz, 1H) 7.35 (d, *J* = 8.8 Hz, 1H), 7.80 (d, *J* = 9.3 Hz, 1H), 7.85 (s, 1H), 7.95 (s, 1H), 8.63 (d, *J* = 4.3 Hz, 1H). ^13^C-NMR (DMSO): *δ* 14.7 (CH_3_), 43.9 (CH_2_), 52.1 (CH_2_), 53.5 (CH_2_), 57.7 (CH_2_), 109.1 (CH), 121.8 (C), 125.1 (CH), 126.2 (CH), 128.8 (CH), 132.8 (CH), 134.9 (C), 138.9 (C), 145.0 (C), 150.5 (C), 151.9 (CH), 156.7 (C), . EIMS (probe) 70 eV, *m/z* (rel. int.): 400.6 [M]^+^ (2), 354.5 [M-46]^+^ (8), 260.4 [M-140]^+^ (100); HRMS (CI) *m/z*: calcd for C_19_H_22_ClN_6_O_2_ [M + H]^+^ 401.1487, found 401.1479. Elemental anal. calcd. (%) for C_19_H_21_ClN_6_O_2_: C, 56.93; H, 5.28; N, 20.96. Found (%): C, 56.74; H, 5.25; N, 20.72.

### Biological Activity

### Test organisms

*Entamoeba histolytica* HK-9 strain (ATCC number 30015) was cultured in LYI-S-2 medium supplemented with antibiotics. *Giardia intestinalis* WB strain (ATCC number 30957) was grown in a modified YI-S medium with antibiotics. Both parasites were cultivated in 15-mL screw-capped borosilicate glass tubes containing 13 mL medium. The tubes were incubated on a 15º horizontal slant at (36-37) ºC. Culture maintenance and subculturing was performed as described [22] *Entamoeba* and *Giardia* were harvested from confluent cultures by chilling of the tubes on ice for 5-10 min. to detach cells, followed by centrifugation at 800 x g for 5 min.

### Antiamoebic and antigiardial activity

The antiamoebic and antigiardial activities of the prepared hybrid molecules **8**, **10-a**, **12**, their precursors, and 1:1 mixtures of these precursors were tested as described [23], with some modifications. Due to insolubility in DMSO-aqueous medium, the bioactivity of **10-b** was not determined. Two milligrams of a test compound or a mixture were dissolved in 10 µL of dimethyl sulfoxide (DMSO) and completed with 1 mL growth medium. The solutions were filter sterilized using 0.22 µm syringe filters and the appropriate volumes of the solutions were taken to prepare the concentrations of each compound or reference drug in 15-mL screw-capped borosilicate glass tubes. For each preparation, concentrations of 240, 120, 60, 30, 15, 7.5, 3.5, 1.7, 0.8, 0.4, 0.2, 0.1 µg/mL medium were prepared in a final volume of 15 mL to exclude air from the tube. For the control mixtures of the precursor compounds, 1:1 molar ratios were prepared. Each tube was inoculated with 20,000 cells of the parasite under testing (*Entamoeba* or *Giardia*). Each compound or mixture was assayed in duplicate in each of three independent experiments. In each assay, the appropriate controls were performed, including the one without any compound or mixture and another with metronidazole as the positive control. The caps of *Entamoeba* and *Giardia* tubes were tightly screwed and wrapped with Parafilm. The tubes were incubated on a 15º horizontal slant at 36-37 ºC for 72 hours.

The parasites in each tube were counted using the standard hemacytometer at the 10X objective. In each count, trypan blue was employed to distinguish live from dead parasites [24]. To permit detachment of *Entamoeba* and *Giardia*, the tubes were placed on ice for few minutes and the parasites were then centrifuged at 1000 x g for 10 min. The supernatant was discarded and 1 mL fresh medium was added to each tube. The final suspension was prepared by mixing 25 µL of the parasite suspension in each tube with 100 µL of 0.4 % trypan blue in phosphate buffered saline (PBS). The 50% inhibitory concentration (IC_50_) was employed as a parameter for biological activity. The IC_50_ is the concentration of compounds which cuts the number of parasites to half that in the negative control (growth medium + DMSO + parasites).

### Cytotoxicity assay

The cytotoxicity of the hybrid compounds and the reference drug, metronidazole, was investigated on Hep-2 and Vero cells using the standard cytotoxicity assay and the trypan blue exclusion method. A cell suspension (10^5 ^cells/mL RPMI medium-10% inactivated foetal calf serum) was prepared from confluent cultures and 100 µL portions of the suspension were added to the wells of 96-well plates. The cells were incubated for 24 h at 37 ºC and 5 % CO_2_ and the medium in each well was then replaced with fresh 150 µL medium. Solutions of the compounds or the reference drug were prepared and sterilized as described in “Antiamoebic and antigiardial activity” section above. Then, 150 µL-two fold serial dilutions of each of the compounds and the reference drug starting at a concentration of 2000 µg/mL in culture medium were prepared in the plates. After 48 hour incubation at 37 ºC and 5 % CO_2_, the number of cells in each well was determined as follows: The medium in each well was gently replaced with 100 µL of 0.25% (w/v) trypsin-0.53 mM EDTA solution and the plates were incubated for 5 min. at 37 ºC to allow cell detachment. Two-hundred microliters of trypan blue solution were added to each well and the plates were, then, placed on ice. The cells were counted in a hemacytometer at the 10X objective. Each compound was assayed in duplicate in each of three independent experiments. In each assay the negative controls (without any compound or reference drug) were included in duplicates. 

## Conclusions

In conclusion, we have described the synthesis of three new hybrid drugs from precursors with known antiparasitic activity. Bioassay of these compounds indicated significant antiparasitic activities against *Entamoeba histolytica* and *Giardia intestinalis* that they could be used as lead structures for the development of antiparasitic drugs. The IC_50_ of the hybrid molecule **12** was found to be about five times less than that of the standard drug metronidazole against those parasites and could be considered as a good drug candidate to be tested against metronidazole-resistant parasites and possibly anaerobic bacteria.
